# Vaginal Ovariotomy

**Published:** 1876-11

**Authors:** Clifton E. Wing


### Vaginal Ovariotomy.

By Clifton E. Wing, M. D.

On February 10, 1876, I saw with Dr. Spalding, of Lowell (the physician of the patient), and my partner, Dr. Warner, a lady whose history was as follows: She was thirty-two years old, unmarried, and was never very strong. The menses began at fourteen, were always regular, but accompanied by considerable pain. Twelve years ago, at the age of twenty, after lifting a heavy weight she began to suffer pains in the back and across the lower part of the abdomen, which became much worse during the monthly period, and had so increased that for the last six years the patient had been obliged to resort to the use of morphine, and, at times, to etherization, when unwell. Five years ago a local examination showed a retroverted uterus, for which passaries were tried without much result, and a year later a body was felt in Douglas's cul-de-sac, which was thought at the time, by Dr. H. R. Storer, who saw the patient, to be ovary. For two years the patient was confined to her room, and part of the time to her bed, by her sufferings, but for the last year or two she has been better, and able to go about a little. She is habitually constipated, and defæcation, even after the use of enemata, is very painful. Micturition frequent, appetite poor, no febrile symptoms, patient emaciated and is in a miserable condition.

An elastic but not distinctly fluctuating mass was felt in Douglas's cul-de-sac, pressing the uterus forward towards the pubes. The fundus uteri was turned somewhat backward, but movable with the sound. The latter entered to the normal depth. With the patient under ether the tumor could not be pressed frem its position, and previous efforts in the same direction had failed. Examination per rectum showed that the mass pressed the bowel against the sacrum, and explained the constipation and painful defæcation.

An aspirator needle was passed into the mass from the vagina, but only a small amount of dark bloody fluid was withdrawn. Dr. E. G. Cutler kindly examined this and reported as follows: "The fluid (about two drahms in quantity) contained a large percentage of albumen. Under the the microscope many red blood corpuscles were seen which had lost their characteristic shape and had become irregular crenated. The surfaces of many showed a few spots not unlike small globules of fat [?]. In addition were seen numerous granular cells in various stages of fatty degeneration, varying in size from that of a white corpuscle to double that size. Hæmatin crystals; no others. Diagnosis fluid, old hæmorrhagic effusion."

The tapping was not followed by any symptoms.

March 30th. The tumor remaining the same, the aspirator was again used, and this time several ounces of the same fluid were removed, and the mass much diminished in size. This second tapping was not followed by improvement; on the contrary, some time afterwards the patient began having occasional slight chills followed by fever, nausea, had headaches; in fact, a mild septicæmia. She continued to lose strength and appetite, though not confined to bed.

When next seen, April 19th, an examination showed the mass behind the uterus to be as large as ever, aud more tense and cystlike. Upon passing an exploring needle from the vagina, as before, a few drops of exceedingly offensive matter escaped. It was evident that the fluid must be thoroughly evacuated, whether it came from an old hæmatocele or from hæmorrhagic ovarian cyst, one of which seemed to be present.

With the aid of Sim's speculum and position, the parts being well drawn down towards the vulva with tenacula, and care being taken to avoid wounding the rectum, an opening was made through the upper part of the vagina and the peritoneum into Douglas's cul-de-sac. Passing my finger through this I distinctly made out a small ovarian cyst, about the size of an orange, fixed in the cul-de-sac by some loose adhesions which easily gave way before my finger. The opening having been enlarged, the cyst was seized with strong forceps, opened and evacuated, then twisted to diminish its size, and pulled through into the vagina. There was no proper pedicle, but the uterus, tipping backward, allowed the broad ligament with the Fallopian tube to come well into the vagina. I intended here to apply a ligature and cut away the cyst, but Dr. Warner in making a digital examination, finding the attachments loose, attempted enucleation with his finger, succeeded in separating the tumor, and brought it away.

There was some bleeding, which soon ceased entirely, and as the uterus went forward the broad ligament slipped back into the abdominal cavity. No ligature whatever was used, but, a coil of small intestine appearing at the opening, I inserted three silk sutures and closed the wound sufficiently to prevent hernia, but left room to pass a catheter, should there be any collection to wash out. The patient was then put to bed.

April 20th. Patient came out of the ether well; had no shock, and passed a good night with the aid of morphine. Reports no more pain than after the previous tapping. No bleeding, some slight chills, no nausea. Taking mild nourishment. Occasional shooting pains in pelvic region. Pulse 116 and good.

April 21st. Pulse 114, temperatuie $100\frac{3}{4}°$ in axilla. Some abdominal distention and pain, and considerable tenderness, but patient moves limbs without much increase of pain. Thirsty, no chills, but nurse reports sweating. Tongue coated, and brown at edges. No appetite, but takes her food. Washed out cul-de-sac, using double catheter and getting away an ounce of fœtid fluid.

April 22d. Pulse 96, temperature $99\frac{1}{2}°$, respiration 18. "Feeling better." Washed out more fœtid fluid. Menses appeared to-day, one week early.

April 23d. Pulse 96, temperature $100\frac{3}{4}°$. Poor night, with pain in lower abdomen, but better to-day.

April 24th. Pulse 96, temperature $99\frac{1}{2}°$. Patient reports herself nicely. In washing out cul-de-sac, which is done daily, some sloughy tissue came away to-day.

April 25th. Pulse aud temperature normal.

April 29th. Bowels have been moved twice by enemata. Pure pus came from wound, which is granulating. Remove the sutures. Catheterization of the bladder, for a time necessary, now dispensed with. To have vaginal injections in place of washing out with catheter.

May 6th. Touched granulations with caustic.

May 18th. Wound closed. Patient walking about the house, and dismissed.

Her rapid recovery was due in great measure to careful watching and faitfhful attendance on the part of Dr. Spalding after the operation.

I met the lady, for the first time since May, a day or two ago. She was no longer using morphine, and was increasing in weight and strength. The menses had been regular since the operation except within the last two months, when the flow had appeared every three weeks. This she attributed to the fact that, through illness in the family, the cares of the household had devolved upon her, and, as she expressed it, she had been obliged to be on her feet and running up and down stairs from morning until night. To the same cause she attributed a backache which she had for a week or more, having been previously free from it.—*Boston Med. and Surg. Journal.*

